# Chemical Composition and Antioxidant Activities of Three Polysaccharide Fractions from Pine Cones

**DOI:** 10.3390/ijms131114262

**Published:** 2012-11-05

**Authors:** Ren-Bo Xu, Xin Yang, Jing Wang, Hai-Tian Zhao, Wei-Hong Lu, Jie Cui, Cui-Lin Cheng, Pan Zou, Wei-Wei Huang, Pu Wang, Wen-Jing Li, Xing-Long Hu

**Affiliations:** 1 School of Food Science and Engineering, Harbin Institute of Technology, 73 Huanghe Road, Nangang District, Harbin 150090, China; E-Mails: xurenboelephant@163.com (R.-B.X.); zhaoht9999@163.com (H.-T.Z.); lwh@hit.edu.cn (W.-H.L.); cuijie2006@163.com (J.C.); ccuilin@hit.edu.cn (C.-L.C.); zoupan0601@163.com (P.Z.); 15146748480@126.com (W.-W.H.); wangpu1237@126.com (P.W.); taoqixiaohai1987@163.com (W.-J.L.); long_0302@163.com (X.-L.H.); 2 Key Laboratory of Agro-product Quality and Safety, Institute of Quality Standard & Testing Technology for Agro-Product, Chinese Academy of Agricultural Sciences, No.12 Zhongguancun South Street, Haidian District, Beijing 100081, China; 3 Key Laboratory of Agrifood Safety and Quality, Ministry of Agriculture, No.12 Zhongguancun South Street, Haidian District, Beijing 100081, China

**Keywords:** *Pinus*, pine cone polysaccharide, GC-MS, antioxidant activity

## Abstract

The traditional method of gas chromatography-mass spectrometry for monosaccharide component analysis with pretreatment of acetylation is described with slight modifications and verified in detail in this paper. It was then successfully applied to the quantitative analysis of component monosaccharides in polysaccharides extracted from the pine cones. The results demonstrated that the three pine cone polysaccharides all consisted of ribose, rhamnose, arabinose, xylose, mannose, glucose and galactose in different molar ratios. According to the recovery experiment, the described method was proved accurate and practical for the analysis of pine cone polysaccharides, meeting the need in the field of chemical analysis of *Pinus* plants. Furthermore; the chemical characteristics, such as neutral sugar, uronic acids, amino acids, molecular weights, and antioxidant activities of the polysaccharides were investigated by chemical and instrumental methods. The results showed that the chemical compositions of the polysaccharides differed from each other, especially in the content of neutral sugar and uronic acid. In the antioxidant assays, the polysaccharide fractions exhibited effective scavenging activities on ABTS radical and hydroxyl radical, with their antioxidant capabilities decreasing in the order of PKP > PAP > PSP. Therefore, although the polysaccharide fractions had little effect on superoxide radical scavenging, they still have potential to be developed as natural antioxidant agents in functional foods or medicine.

## 1. Introduction

The genus *Pinus* (family *Pinaceae*) is one of the most widely distributed genera of trees in the Northern hemisphere, encompassing nearly 100 species [1]. Many Pinaceae have been planted as windbreaks and for protection against tides and sand, due to their high tolerance to dryness, salt air and cold wind, and have been used for fuel, materials, resins, and seeds, *etc*., for many years [2]. Nowadays, extracts from different parts of *Pinus* (bark, needle, cone and resin) have been used in many areas for their high biological and antioxidant activities. It has been reported that pine bark extract has an anti-proliferation effect on human breast cancer cells, and shows strong DPPH radical scavenging activity, reducing power, nitric-oxide scavenging activity and ferrous-ion chelating ability [3,4]. A substance extracted from pine needles of *Pinus morrisonicola* has been found to be more effective than ascorbic acid in scavenging DPPH radicals and can be applied in the development of alcoholic and vinegar products [5]. Recently, significant attention has been paid to the polysaccharides extracted from pine cones, which have the potential to be used as novel antioxidants. An acidic polysaccharide fraction was isolated from pine cones of *Pinus parviflora Sieb. Et Zucc* and showed various pharmacological activities, such as antitumor, anti-microbial, anti-HIV, and stimulation of tumor necrosis factor (TNF) production [6].

Various compounds have been extracted from pine cones, such as polysaccharide, lignin-related compounds and essential oils, among which the content of polysaccharide accounts for about 50% (w/w) [7,8]. Several analytical methods used to determine the monosaccharide composition of polysaccharides have been reported, such as gas chromatography (GC) and high-performance liquid chromatography (HPLC). GC, as a well-established technique, has been widely used for carbohydrate profiling, especially for detecting carbohydrate monomers in complex matrices because of its high sensitivity and good selectivity. Classical derivatization methods before GC analysis are needed to increase volatility of monosaccharides and decrease interaction with the analytical system, mainly including trimethylsilyl (TMS), aldononitrile acetate and alditol acetate procedures [9]. TMS, the most commonly used method, often results in a complex chromatographic pattern owing to anomerization, whereby the α- and β-anomers of the pyranoside and furanoside forms of the monosaccharide can produce multiple peaks [10]. Aldononitrile acetate and alditol acetate methods have been utilized for monosaccharide component analysis in both neutral and amino sugars. Aldononitrile acetate is usually formed by two-step derivatization procedure and lack of reproducibility for some aminosugars [11]. Compared with the two methods described above, alditol acetates can be formed in a one-step reaction and, once formed, are stable allowing post-derivatization cleanup and storage of treated samples for extended periods. In addition, acetylation of alditols eliminates the anomeric center and therefore simplifies the chromatograms dramatically, as most sugars produce one chromatographic peak [12]. Even though the alditol acetates method has been used for analysis of monosaccharide components for a long time, it was mostly employed for determination of carbohydrate profiles of bacterial sugars, for trace detection of bacteria or their constituents in complex clinical or environmental matrices, or to determine their physiological status [13–15], and seldom used in analysis of plant polysaccharides. However, this derivatization method was earlier investigated in the field of wood for analysis of sugar mixtures resulting from the hydrolysis of southern pine wood pulps [16]. Therefore, we tried to apply this method to analyze polysaccharides from *Pinus* and verified its accuracy.

Previously, we focused our attention on the chemical constituents of cones of coniferous tree, and isolated diterpenes from pine cones of *P. armandii* and *P. koraiensis*. Furthermore, the essential oils from pine cones of three *Pinus* were also investigated in our previous work [17–21]. In this paper, apart from investigating the precision of the GC-MS with alditol acetates derivatization method on monosaccharide composition analysis of pine cone polysaccharides, the experiment was conducted to analyze the chemical composition of polysaccharides isolated from pine cones of three *Pinus* species, and then compare their differences in antioxidant activities. All the information provided here will contribute to better utilization of materials from pine cones as novel medical plant products.

## 2. Results and Discussion

### 2.1. Extraction, Purification and Physicochemical Properties of Polysaccharide Fractions

**Figure 1:**
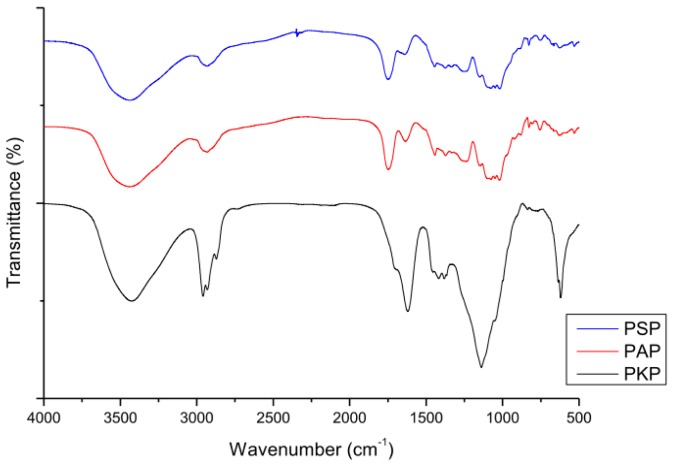
FTIR spectra of the three polysaccharides.

According to the procedure as described in the experimental section, three polysaccharide fractions were successfully isolated from pine cones of *P. koraiensis*, *P. armandii* and *P. sylvestris var. mongolica* by water extraction and ethanol precipitation, and then purified by trichloroacetic acid and activated carbon. The three purified polysaccharide fractions were coded as PKP, PAP and PSP and their physicochemical properties, such as neutral sugar content, uronic acid content, molecular weight, and amino acid composition are given in Table 1. It can be seen in Figure 1 that PKP, PAP and PSP all displayed a broad stretching intense characteristic peak near 3430 cm^−1^ due to hydroxyl stretching vibration of the polysaccharide fractions, and a weak C–H stretching vibration band in the region of 2930 cm^−1^. The band towards 1750 cm^−1^ was attributed to stretching vibration of C=O in the protonated carboxylic acid, which resulted from the presence of uronic acids. Furthermore, the band at 1635 cm^−1^ was due to the bound water.

### 2.2. Analysis of Monosaccharide Composition by GC-MS

#### 2.2.1. Validation of the Method Developed

**Figure 2:**
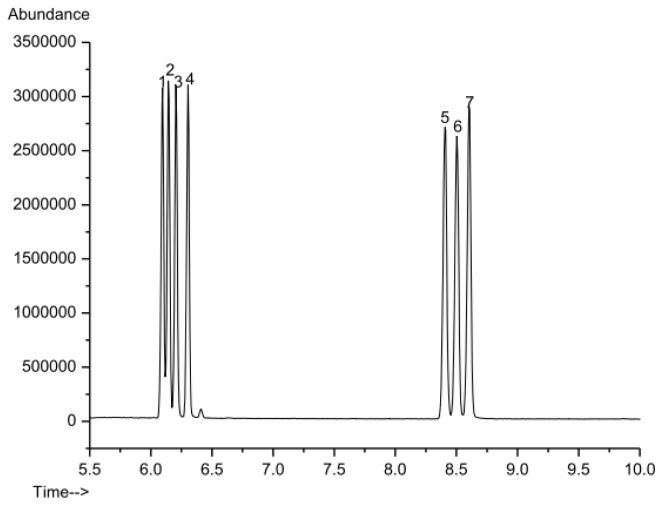
GC-MS chromatogram of 7 standard monosaccharides. (**1**) d-ribose; (**2**) l-rhamnose; (**3**) l-arabinose; (**4**) d-xylose; (**5**) d-mannose; (**6**) d-glucose; (**7**) d-galactose.

The GC-MS method was validated in terms of linearity and limit of detection (LOD). A standard solution including seven kinds of standard monosaccharide (d-ribose, l-rhamnose, l-arabinose, d-xylose, d-mannose, d-glucose and d-galactose) was prepared and the calibration curves were obtained by analyzing five points (0.001–0.400 mg/mL) according to the derivatization procedure described in the experimental section. The results demonstrated that the seven monosaccharide derivatives could be well separated under the established GC-MS system (Figure 2) and the linear regression parameters of the calibration curves are shown in Table 2. The good linearity (linear regression coefficients *R*^2^ > 0.998) of the MS detector response between *y* (peak area of monosaccharide derivative) and *x* (concentration of the standards) was found for all monosaccharide derivatives in the tested range. Furthermore, LOD of each monosaccharide derivative was determined to be in the range of 0.45–0.50 μg/mL (Table 2) by comparison of peak height with baseline noise level (S/N = 3), which indicated that the selectivity and sensitivity of the method were satisfactory.

#### 2.2.2. Application to the Analysis of Three Polysaccharide Fractions

**Figure 3:**
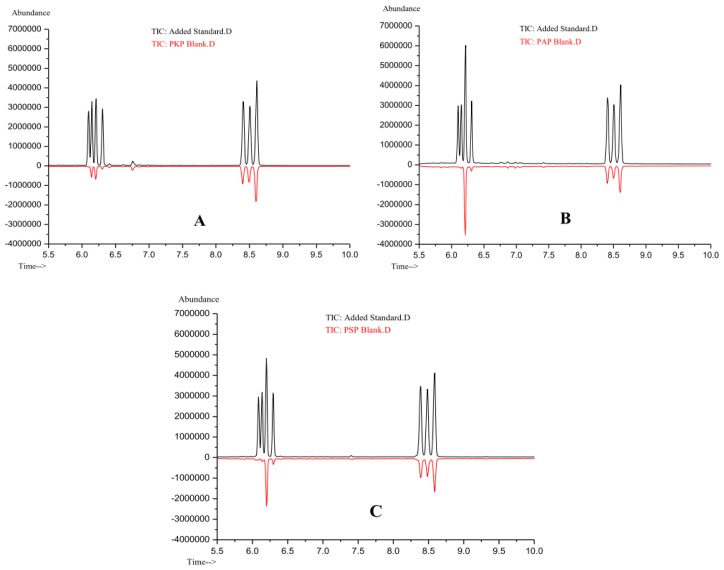
GC-MS chromatogram of samples at 0.200 mg/mL spiked level and the blank polysaccharide matrix. (**A**) PKP; (**B**) PAP; (**C**) PSP.

In order to evaluate the applicability of the proposed method, the polysaccharide extract was hydrolyzed with TFA and derivatized using the experimental procedure described above. The released monosaccharide derivatives were analyzed by the described GC-MS method under optimized conditions. Figure 3 shows the chromatograms of component monosaccharide derivatives of PKP, PAP and PSP. By comparing their typical chromatograms with Figure 2, the component monosaccharides could be identified and the results were listed in Table 3. The results indicated that PKP, PAP and PSP were all heteropolysaccharides and consisted of d-ribose, l-rhamnose, l-arabinose, d-xylose, d-mannose, d-glucose and d-galactose. However, their corresponding molar contents were different from each other. It could be seen that the molar ratios of monosaccharide components in PAP and PSP were similar and the predominant monosaccharide in them was l-arabinose, whereas it was d-galactose in PKP. The molar content of d-ribose was the least in all three polysaccharide fractions.

Additionally, a recovery experiment was performed in order to investigate the accuracy of this method. Different concentrations of standard solution were added to the samples and the resulting spiked sample was subjected to the entire analytical sequence. Each spiking level was assessed in three repetitions. The precision of the method was described as the value of relative standard deviation (RSD) and recoveries were calculated based on the difference between the total amount determined in the spiked sample and the amount observed in the non-spiked sample. The results showed that the recoveries of all the seven monosaccharides in PKP, PAP and PSP ranged between 65.40% and 110.40% and RSD values fell within 0.4%–20.40% (Table 4). Such results further demonstrated that this method was practical for the analysis of the polysaccharide fractions from pine cones of *Pinus*.

Alditol acetate approach, as the traditional method of derivatization before GC-MS analysis, has been used for many years, but, to the best of our knowledge, up to now, there were scarcely any reports on its accuracy of the monosaccharide component analysis using external standard method as discussed in detail in this paper. Although a large number of manual processing steps were needed which made the procedure tedious to perform, the advantages of the proposed GC-MS method was still obvious because of its applicability, sensitivity and selectivity. From the results, we could conclude that the established GC-MS method of alditol acetates was applicable to the monosaccharide component analysis of the three polysaccharide fractions extracted from pine cones of *Pinus*, being precise, selective, and sensitive, with potential for accurate monosaccharide component analyses for the other *Pinus* species.

### 2.3. Antioxidant Activity

#### 2.3.1. Scavenging Effects on ABTS Radicals

**Figure 4:**
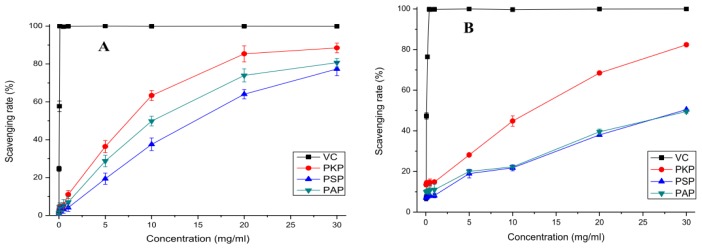

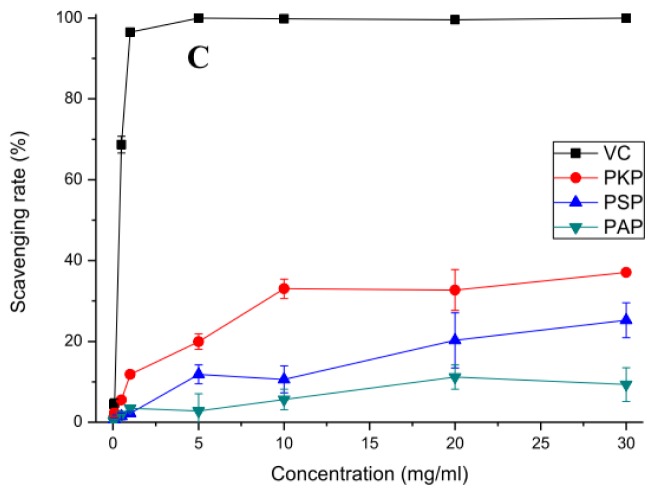
The *in vitro* antioxidant activity of the three polysaccharides from pine cones. (**A**) The ABTS radical scavenging activity; (**B**) The hydroxyl radical scavenging activity; (**C**) The superoxide radical scavenging activity.

The model of scavenging the stable ABTS radical is a widely used method for evaluating total antioxidant power of single compounds and complex mixtures of various plants [22]. The ABTS radical-scavenging ability of PKP, PAP and PSP is shown in Figure 4A with ascorbic acid as the positive control. It was clearly observed that the polysaccharide fractions all had significant potent scavenging ability on ABTS radicals in a concentration-dependent manner from 0.01 to 30 mg/mL and their scavenging ability decreased in the order of PKP > PAP > PSP. When the concentration reached 30 mg/mL, all polysaccharide fractions showed a strong effect on ABTS radical with a scavenging rate of above 70%. However, as presented in Figure 4A, the scavenging ability of ascorbic acid on ABTS radical was much better than PKP, PAP and PSP.

#### 2.3.2. Scavenging Effects on Hydroxyl Radicals

Among the reactive oxygen species, the hydroxyl radical is the most reactive one which can easily cross cell membranes, readily react with most biomolecules including carbohydrates, proteins, lipids, and DNA in cells and induce severe tissue damage or cell death [23]. Thus, removing hydroxyl radicals is important for the protection of living systems. Figure 4B shows the percentage hydroxyl radical scavenging effects of PKP, PAP and PSP at different concentrations (0.05–30 mg/mL). At the test concentrations, the increase in concentration of the polysaccharide fractions was synonymous to an increase in scavenging capacity. The radical-scavenging abilities of PAP and PSP were nearly the same, but much lower than that of PKP. At 30 mg/mL, the scavenging rates of PKP, PAP and PSP were 82.37%, 49.52% and 50.49%, respectively, which showed that PKP had the most significant ability on scavenging hydroxyl radicals among the polysaccharide fractions. Furthermore, ascorbic acid, as the positive control, exhibited a more effective scavenging activity on hydroxyl radicals than the polysaccharide fractions. There are two types of antioxidant mechanisms for hydroxyl radical scavenging: one may be due to the combination of radicals and the hydrogen supplied by polysaccharides to form a stable radical, which terminates the radical chain reaction. The other possibility is that the radical ions which are necessary for radical chain reaction are chelated by polysaccharides, and consequently the reaction is terminated. However, the exact mechanism is still not fully understood [24].

#### 2.3.3. Scavenging Effects on Superoxide Radicals

Although superoxide is regarded as a weak oxidant, in most organisms, it can degrade continuously and form other reactive oxygen species such as hydrogen peroxide and hydroxyl radical through dismutation and other types of reaction *in vivo*. The superoxide radical and its derivatives could trigger peroxidation of lipids, and then induce pathological incidents such as arthritis and Alzheimer’s disease, which is extremely harmful to human beings [25,26]. From Figure 4C, we can see that PKP, PAP and PSP had little effects on scavenging superoxide radical. At the highest test concentration, their scavenging rates were lower than 40%, which were far lower than that of ascorbic acid. The result revealed that the polysaccharide fractions had little scavenging abilities on superoxide radicals. The mechanism of scavenging may be associated with dissociation energy of O–H bond, that is, the higher the number of electron withdrawing groups attached to polysaccharide, the weaker the dissociation energy of O–H bond [27]. PKP, PAP and PSP had scarcely effective scavenging ability on superoxide radicals, which may be due to the absence of enough strong electrophilic groups.

In this experiment, the polysaccharide fractions showed different degrees of antioxidant effects. Generally, the antioxidant activities of polysaccharides are supposed to relate to the chemical characteristics such as molecular weight, monosaccharide composition and configuration. Different monosaccharide compositions of polysaccharides may contribute to different bioactivity. The monosaccharide in the polysaccharide, as the reductive agent, could supply hydrogen to combine with radicals and form a stable compound to terminate the radical reaction [24]. Also, different monosaccharide compositions lead to different degrees of backbone and branch formations of the polysaccharides, which may be responsible for their different antioxidant activities [28]. It has been reported that the antioxidant properties of the polysaccharides depended on the ratio of different monosaccharide in the composition, among which rhamnose was the most significant factor associated with antioxidant properties [29]. According to the analysis of monosaccharide components among the polysaccharides, PSP and PAP shared similar monosaccharide components, which were obviously different from PKP, and the higher content of rhamnose in PKP (Table 3) might be a possible explanation for its higher antioxidant activity. According to other researches, the presence of uronic acids in polysaccharides usually has a key impact on their antioxidant activities and higher content in uronic acids is consistent with stronger radicals scavenging ability [30]. On the one hand, their electron-withdrawing carboxyl groups could activate the hydrogen atom of sugar residues through field and inductive effects [31]. Furthermore, it was reported that the compounds with structures containing two or more of the following functional groups: -OH, -SH, -COOH, -PO_3_H_2_, C=O, -NR_2_, -S- and -O- in a favorable structure-function configuration could chelate metals and prevent the generation of the hydroxyl radical [32]. In the hydroxyl radical scavenging assay, the higher scavenging ability of PKP and PAP on hydroxyl radical might be due to their higher content of uronic acid. Another factor contributing to high biology activity is molecular weight and a relatively low molecular weight of polysaccharides appeared to increase the antioxidant activity [33]. Apart from the factors mentioned above, the proteins and polyphenolic compounds in the polysaccharide also played an important role in the bioactivity of polysaccharides, and it has been reported that the presence of proteins and polyphenolic compounds in the polysaccharides contributed to the higher antioxidant activity [34,35]. The polyphenolic compounds in the polysaccharides were determined by the Bonvehi’s method [36]. The results showed that both proteins and polyphenolic compounds in the polysaccharides amounted to about 1%, which is so little compared with polysaccharides that they could hardly account for the antioxidant activities exhibited by the polysaccharides. Further detailed information is needed to elucidate the structure-function relationship of polysaccharides with regard to its antioxidant activity.

## 3. Experimental Section

### 3.1. Materials and Reagents

*P. koraiensis*, *P. armandii* and *P. sylvestris var. mongolica* cones were collected in Heilongjiang Province and Yunnan Province of the People’s Republic of China, respectively. The samples were collected just at the time of maturity. After cutting into small pieces, the air-dried cones were ground into powders. d-xylose, l-arabinose, d-glucose, d-galactose, d-mannose, d-ribose, and l-rhamnose were purchased from Tianjin Kermel Chemical Reagent Co. (Tianjin, China). Trifluoroacetic acid (TFA), acetic anhydride, pyridine, acetic acid, and 2,2-azino-bis(3-ehylbenzthiazoline-6-sulphonate) (ABTS) were purchased from J & K Technology Company (Harbin, China). All the other chemicals used in the work were of analytical grade.

### 3.2. Extraction and Purification of Pine Cone Polysaccharide

The crude polysaccharides from 3 kinds of pine cones were isolated by hot-water extraction and ethanol precipitation according to the method by Sun with a few modifications [37]. Briefly, prior to extraction, the pine cone materials (200 g) were extracted with 95% ethanol for 2 h to remove aliphatic compounds. The dried residue was extracted with distilled water (1:12, *w*/*v*) at 100 °C for 4 h, and then the aqueous extracts were collected, concentrated to 150 mL, and precipitated with 1200 mL of 95% ethanol at room temperature for 8 h. After that, the precipitate was dissolved in distilled water, and the 0.5% (*w*/*w*) crude polysaccharide solution was mixed with 10% trichloroacetic acid (1:1, *v*/*v*) to remove the protein. After staying at room temperature for 10 h, the supernatant was collected and bleached by activate carbon. For this purpose, 1.5% activate carbon was added to the 0.5% crude polysaccharide solution (pH adjusted to 3), and the solution was shaken vigorously at 50 °C for 60 min. Finally, the supernatant was collected, concentrated, and kept at 4 °C for subsequent analysis.

### 3.3. Molecular Weight Determination

The purified polysaccharides dissolved in ultrapure water (final concentration was 1 mg/mL) were characterized by gel-permeation chromatography (GPC, Agilent Technologies, Santa Clara, CA, USA), using Agilent 1100 instrument (Agilent Technologies, Santa Clara, CA, USA) equipped with Agilent Technologies PL aquagel-OH Mixed column (Agilent Technologies, Santa Clara, CA, USA) and Agilent G1362A Refractive index Detector (Agilent Technologies, Santa Clara, CA, USA). The linear regression was calibrated with Dextrans 106, 194, 620, 1470, 4,120, 11,840, 25,820, 58,400, 124,700, 460,000, 965,000, 1250 and 450. 50 μL samples were injected by Agilent G1313A Autosampler into the gel column, then eluted with ultrapure water at 25 °C and a flow rate of 1.0 mL/min. The molecular weight (*M*w) was obtained from the calibration curve. All samples were filtrated through a 0.45 μm pore diameter membrane prior to analysis.

### 3.4. Analysis of Monosaccharide Composition by GC-MS

#### 3.4.1. Hydrolysis of the Pine cone Polysaccharide Fraction

Hydrolysis was carried out with TFA. The purified polysaccharide sample (15 mg) was hydrolyzed with 4 mL of 2 M TFA at 110 °C for 4 h in a sealed glass tube. After hydrolysis, the solution was evaporated to dryness at 50 °C, and then a stream of nitrogen and methanol (3 mL) were used to remove the excess acid. This procedure was repeated 5 times to remove the TFA completely. After that, the hydrolyzed products were ready for the following derivatization.

#### 3.4.2. Derivatization

A reducing reaction must be carried out before derivatization, through which the aldoses in the standard solution or in the hydrolyzed sample are reduced to their corresponding alditols. The reducing reaction was performed at room temperature for 3 h by adding 25 mg sodium borohydride. Several drops of glacial acetic acid were added to stop the reaction until air bubbles disappeared and then the solution was evaporated to dryness by rotary evaporator at 50 °C. Methanol (3 mL) and a stream of nitrogen gas were used to remove the reducing agent for 5 times, and then the residue was dried at 110 °C for 15 min to remove the moisture. The acetylation was carried out with acetic anhydride (3 mL) and pyridine (1 mL) in a water bath at 100 °C for 5 h. After that, the mixture was evaporated to dryness at 85 °C and trichloromethane (5 mL) was added to dissolve the residue. The organic phase was washed with distilled water (5 mL) for 5 times to remove the impurities. Finally, the water was removed with anhydrous sodium sulfate and the organic phase was transferred into a GC vial for GC-MS analysis.

#### 3.4.3. GC-MS Analysis

The GC-MS was used for separation of monosaccharides. A capillary column DB-5 (60 m × 0.25 mm I.D., 0.25 μm film thickness) was used, with helium as carrier gas at a constant flow of 1 mL/min. The temperature program was the following: initial temperature 200 °C, 25 °C/min ramp to 250 °C, and held for 10 min. The total analysis time was 12 min and the equilibration time 2 min. The temperature of the injection port was 250 °C and a 1 μL volume was injected in splitless mode. The mass spectrometer was operated in electron ionization mode with an ionizing energy of 70 eV, ion source temperature 230 °C, MS Quad temperature 150 °C, electron multiplier voltage (EMVolts) 1750 V when performing selected ion monitoring, scanning from m/z 50 to 500.

### 3.5. Neutral Sugar, Uronic Acid and Amino Acid Analysis

The Neutral sugar contents in polysaccharides were determined by the phenol-sulphuric acid method [38] with d-glucose as the standard at 490 nm. Uronic acid contents were determined by photometry with m-hydroxybiphenyl at 525 nm with d-galacturonic acid as the standard [39]. Amino acids were released by hydrolysis with 6 M HCl at 110 °C for 22 h in a sealed tube according to a previously described method [40].

### 3.6. FT-IR

The structural characteristics of the polysaccharide sample were recorded on a Fourier-transform infrared spectrophotometer (Perkin-Elmer Instruments, Norwalk, CT, USA). The sample was ground with KBr powder (spectroscopic grade) and then pressed into 1 mm pellet for FT-IR measurement in the frequency range 4000–500 cm^−1^[41].

### 3.7. Antioxidant Activity

#### 3.7.1. ABTS Radical Scavenging Assay

The radical scavenging activity of the polysaccharides against radical cation (ABTS·^+^) was measured using an improved method as described by Re and others [42]. The ABTS radical cation solution was prepared through the reaction of 7 mM ABTS and 2.45 mM potassium persulfate. After incubation at 23 °C in the dark for 16 h, the ABTS·^+^ solution was diluted with distilled water to obtain an absorbance of 0.70 ± 0.02 at 734 nm before use. Reaction mixture consisted of 3.8 mL ABTS·^+^ solution and 0.2 mL polysaccharide solution of different concentrations (0.01–30 mg/mL) and then the mixture was left standing at room temperature for 6 min, after which the absorbance at 734 nm was immediately recorded. Ascorbic acid was used as the positive control. The scavenging activity was calculated using the following Equation 1:

(1) $$\text{Scavenging rate }(\%)=[1-(A_{1}-A_{2})/A_{0}]\times 100$$

where *A*_0_ is the absorbance of the control group (without polysaccharides), *A*_1_ is the absorbance of the test group and *A*_2_ blank is the absorbance of samples only (without ABTS·^+^).

#### 3.7.2. Hydroxyl Radical Scavenging Assay

The scavenging capacity of the polysaccharides on hydroxyl radicals was evaluated according to the reaction of salicylic acid and residual hydroxyl radicals. Hydroxyl radical scavenging assay was performed according to the method of Smirnoff and others [43] with a few modifications. Hydroxyl radicals were generated by the Fenton reaction. The reaction mixture (4.0 mL) containing 1 mL FeSO_4_ (9 mM), 1 mL H_2_O_2_ (8.8 mM), 1 mL of various concentrations (0.05–30 mg/mL) of polysaccharide solution and 1 mL salicylic acid (9 mM) was incubated at 37 °C for 1 h and then the absorbance was recorded at 510 nm. Ascorbic acid was used as the positive control. The scavenging activity was calculated using the following Equation 2:

(2) $$\text{Scavenging rate }(\%)=[1-(A_{1}-A_{2})/A_{0}]\times 100$$

where *A*_0_ is the absorbance of the control group (without polysaccharide), *A*_1_ is the absorbance of the test group and *A*_2_ is the absorbance without salicylic acid.

#### 3.7.3. Superoxide Radical Scavenging Assay

Superoxide radical was generated in the system of pyrogallol’s autoxidation in an alkalescent condition. The assay was performed according to a previous method [44] with slight modifications. Briefly, 0.2 mL sample solution of polysaccharides at different concentrations (0.05–30 mg/mL) was mixed with 5.7 mL Tris-HCl buffer (50 mM, pH 8.20) and then the mixture was incubated at 25 °C for 20 min. After that, 0.1 mL pyrogallol solution (6 mM) was added to the mixture quickly and the absorbance of the reactive solution was measured immediately at 320 nm every 30 s. The curve was made based on the absorbance value and the absorbance of the mixture at 6 min was recorded. Ascorbic acid was used as the positive control. The scavenging activity was calculated using the following Equation 3:

(3) $$\text{Scavenging rate }(\%)=[1-(A_{1}-A_{2})/A_{0}]\times 100$$

where *A*_0_ is the absorbance of the control group (without polysaccharide), *A*_1_ is the absorbance of the test group and *A*_2_ is the absorbance without pyrogallol.

## 4. Conclusions

In this study, we successfully obtained three purified polysaccharide fractions (PKP, PAP, and PSP) from pine cones of three *Pinus* species. The chemical and GC-MS analytical results demonstrated that PKP, PAP and PSP consisted of Rib, Rha, Ara, Xyl, Man, Glu and Gal in different molar ratios and the uronic acid contents of PKP and PAP were higher than that of PSP. Based on the *in vitro* antioxidant assays, the antioxidant capabilities of the polysaccharide fractions were evaluated and the results indicated that they all presented strong scavenging abilities on ABTS radicals and hydroxyl radicals, with scavenging ability decreasing in the order of PKP > PAP > PSP, but almost had no effect on superoxide radicals. Furthermore, in this experiment, the monosaccharide component analysis of polysaccharides was achieved by GC-MS with pretreatment employing alditol acetates derivatization method, and this method was validated to be sensitive and suitable to analysis of polysaccharides from pine cones of *Pinus*.

## Figures and Tables

**Table 1:** Total sugar contents, uronic acid contents, molecular weight, and amino acid contents of PKP, PSP and PAP.

Component	Polysaccharide		

	PKP	PSP	PAP
Neutral sugar (%)	41.33	33	42
Uronic acid (%)	51.52	37.13	56.32
Molecular weight (kDa)	4186.0	4239.2	4166.1
Amino acid component (%) b			
Asp	nd a	Nd a	0.06
Thr	0.06	0.07	0.12
Ser	0.04	0.05	nd a
Glu	nd a	0.01	0.09
Gly	0.03	0.03	0.14
Cys	0.02	0.05	0.08
Val	0.07	0.08	0.08
Met	nd a	0.07	0.09
Ile	0.01	0.02	0.01
Lei	0.01	0.02	0.04
Tyr	0.01	0.03	0.04
Phe	0.01	0.01	0.04
Lys	0.01	0.01	0.03
His	nd a	0.01	0.01
Arg	nd a	0.01	0.04

a nd = not detected.

b Asp = Asparagine; Thr = Threonine; Ser = Serine; Glu = Glutamic acid; Gly = Glycine; Cys = Cysteine; Val = Valine; Met = Methionine; Ile = Isoleucine; Lei = Leucine; Tyr = Tyrosine; Phe = Phenylalanine; Lys = Lysine; His = Histidine; Arg = Arginine.

**Table 2:** The regression equation and linear regression parameters of seven standard monosaccharides.

Monosaccharide	R.T. (min)	Linear Range (mg/mL)	Regression Equation	*R*^2^	LOD (μg/mL)
d-Ribose	6.05	0.01–0.40	*Y* = 7.88 × 10^3^*X* − 2.15 × 10^5^	0.9998	0.45
l-Rhamnose	6.16	0.01–0.40	*Y* = 6.19 × 10^3^*X* − 1.34 × 10^5^	0.9997	0.45
l-Arabinose	6.21	0.01–0.40	*Y* = 6.42 × 10^3^*X* − 1.12 × 10^5^	0.9999	0.45
d-Xylose	6.32	0.01–0.40	*Y* = 7.01 × 10^3^*X* − 1.20 × 10^5^	0.9999	0.45
d-Mannose	8.41	0.01–0.40	*Y* = 5.31 × 10^3^*X* − 1.79 × 10^5^	0.9988	0.50
d-Glucose	8.51	0.01–0.40	*Y* = 4.60 × 10^3^*X* − 1.06 × 10^5^	0.9997	0.50
d-Galactose	8.61	0.01–0.40	*Y* = 5.51 × 10^3^*X* − 1.09 × 10^5^	0.9998	0.50

**Table 3:** Monosaccharide composition of PKP, PSP and PAP.

Polysaccharide	Monosaccharide (mol%)						

	Ribose	Rhamnose	Arabinose	Xylose	Mannose	Glucose	Galactose
**PKP**	2.16	11.25	13.78	3.84	17.62	17.24	34.11
**PSP**	2.11	2.67	35.54	5.07	15.48	16.61	22.52
**PAP**	1.84	2.05	50.25	4.32	12.60	10.47	18.47

**Table 4:** The Average recoveries and RSD (in parentheses) of 7 monosaccharides in PKP, PSP and PAP (*n* = 3).

Monosaccharide	Average recovery (%)								

	PKP			PSP			PAP		
		
	Spiked level (mg/mL)			Spiked level (mg/mL)			Spiked level (mg/mL)		
		
	0.02	0.10	0.20	0.02	0.10	0.20	0.02	0.10	0.20
d-Ribose	85.00 (11.2)	89.66 (7.8)	90.52 (2.4)	74.55 (4.5)	85.06 (3.3)	89.57 (2.2)	89.05 (7.6)	87.52 (6.0)	88.98 (9.7)
l-Rhamnose	103.80 (13.3)	87.94 (9.9)	86.35 (2.8)	82.8 (2.7)	90.44 (1.6)	90.89 (4.2)	88.55 (20.4)	91.74 (7.2)	82.02 (11.9)
l-Arabinose	97.20 (9.8)	94.44 (3.2)	91.24 (5.3)	110.4 (11.3)	104.94 (3.6)	94.87 (13.2)	100.45 (10.8)	109.74 (6.7)	89.87 (8.0)
d-Xylose	91.10 (5.2)	93.64 (5.4)	92.23 (1.4)	79.25 (7.1)	89.99 (4.8)	91.45 (2.0)	92.25 (1.9)	98.26 (3.9)	92.03 (0.9)
d-Mannose	87.50 (3.4)	89.66 (4.4)	88.68 (3.2)	92.60 (20.7)	93.75 (5.3)	97.35 (3.6)	90.50 (13.6)	94.10 (3.7)	84.12 (3.4)
d-Glucose	96.20 (11.2)	89.00 (10.6)	88.10 (2.4)	93.30 (17.0)	83.55 (1.3)	93.09 (0.7)	91.10 (6.8)	99.85 (2.4)	82.37 (6.9)
d-Galactose	65.40 (14.2)	89.96 (8.5)	87.09 (4.8)	108.05 (16.1)	103.28 (0.4)	95.92 (6.1)	93.70 (13.7)	102.80 (8.6)	88.33 (0.8)
